# MicroRNA Expression Profiling of Lung Cancer with Differential Expression of the RON Receptor Tyrosine Kinase

**DOI:** 10.1155/2021/5670675

**Published:** 2021-09-24

**Authors:** Huilin Ou, Keda Chen, Hongcheng Wu

**Affiliations:** ^1^Ningbo Medical Centre, Li Huili Hospital Affiliated of Ningbo University, Ningbo 315040, China; ^2^Shulan International Medical College, Zhejiang Shuren University, Hangzhou 310015, China

## Abstract

**Background:**

The Ron receptor tyrosine kinase (RON) can act as a protooncogene and may play a prominent role in the initiation and development of lung cancer. microRNAs (miRNA) are master regulators of gene expression through direct or indirect regulation, and impact all aspects of cell biology.

**Methods:**

Nonsmall-cell lung cancer (NSCLC) samples and small-cell lung cancer (SCLC) were stratified based on RON expression to identify miRNA profiles associated with RON expression levels, differentially expressed miRNA regulated by RON were screened out, and their biological behavior was analyzed.

**Results:**

miRNA expression was most significantly affected by cancer type, and we found 85 miRNAs that were significantly differentially expressed between NSCLC and SCLC. There were 46 miRNAs differentially expressed between high RON expressing NSCLC compared to low RON expressing NSCLC. Biological processes and pathways found to be significantly influenced by RON expression included epithelial-mesenchymal transition (EMT) and activation of the PI3K-Akt and MAPK signaling pathways.

**Conclusions:**

These data may provide the basis for a novel strategy to characterize lung cancer by RON expression and microRNA genotyping.

## 1. Introduction

Lung cancer is the leading cause of cancer-related deaths in the world, and mutations and deregulation of many proteins and pathways, including EGFR, ALK, ROS1, and MET, play a significant role in lung cancer occurrence and development [1, 2]. Better identification and utilization of these disease-related biomarkers could lead to improvement in precision treatment of lung cancer, ultimately reducing disease incidence, lowering disease recurrence, and improving survival and the quality of life for lung cancer patients.

RON is located at the chromosome 3p21.3 region, a region that has tumor suppressor activity and undergoes frequent loss of heterozygosity in human lung and breast cancers. The mature RON is a 180 kDa heterodimer composed of a 40 kDa extracellular chain and a 150 kDa transmembrane *β*-chain. Stimulation of RON by its ligand, MSP, promotes invasive growth of epithelial cells, resulting from the integration of a number of input pathways, including epithelial-mesenchymal transition (EMT), cell-cell dissociation (“scattering”), and extracellular matrix growth and invasion [3, 4].

RON is primarily expressed in cells of epithelial origin including lung cells. Several lung cancer cell lines overexpresses Ron and variant isomers, and normal lung tissue exhibits minimal expression of RON compared to adjacent tumor tissue. These data suggest that RON expression may be related to the occurrence and development of both NSCLC and SCLC and could be used as a prognostic indicator for lung cancer patients [5]. Regulation of how RON expression and splicing is deregulated in NSCLC and SCLC requires further characterization. The frequency and intensity of RON and phospho-RON expression in NSCLC is lower than in SCLC, and the *β*-RON isoform (around 150 kDa) was found to be the dominant isoform in NSCLC, compared to the 120 kDa isoform in SCLC. Furthermore, northern blot analysis verified that the RON gene is normally transcribed in the lungs. These data suggest that characterizing mechanisms regulating RON gene expression are of great significance to improve understanding of lung tumor occurrence and development [6].

microRNAs (miRNAs) are small noncoding RNAs, ∼18–25 nucleotides in length, that regulate gene expression at the posttranscriptional level by hybridizing to target mRNAs, and either inhibiting gene translation or promoting degradation of messenger RNAs (mRNA) by miRNAs are thought to regulate at least 30% of human gene expression and are involved in essential biological processes, including cell-cycle control, cell lineage fate decision, cell survival, tissue patterning, vascular development, immune control, and metabolism [7]. miRNAs act as oncogenes or tumor suppressors in various tumors, including lung cancer [8].

RON and miRNAs are closely related to the occurrence and development of lung cancer; however, it is unclear how miRNAs interact with RON to facilitate lung cancer progression. In vitro, RON mutations that alter miRNA expression induce oncogenic and metastatic potential. miRNA profiles are significantly affected by RON expression in pancreatic cancer, indicating the potential role of miRNAs in tumor invasion and metastasis [9]. In the present study, we compare miRNA profiles of lung cancer samples with differential expression of RON in order to investigate how RON regulates miRNA expression in both NSCLC and SCLC.

## 2. Materials and Methods

### 2.1. MicroRNA Expression Analysis

This research has been carried out in accordance with the World Medical Association Declaration of Helsinki and was approved by the Institutional Review Board (CWO) of Medical School of Ningbo University, Ningbo, China (2020-YXY-0035). All subjects provided written informed consent. Nine NSCLC samples and nine SCLC samples were analyzed by miRNA microarray. Each group was classified based on RON expression levels, no expression, low expression, and high expression, with three samples representing each level. miRNA expression patterns were compared between NSCLC and SCLC samples with different RON expression levels. The expression of RON was determined by Western blot and immunohistochemistry.

### 2.2. Immunohistochemistry of Lung Tissue

Fragments of lung tissue were incubated with anti-RON monoclonal antibodies Zt/*f*2 overnight at 4°C, followed by the DAKO Envision DAB System visualization reagents (DAKO, Denmark). All slides were counterstained with hematoxylin. RON expression was assessed using a semiquantitative scoring system.

### 2.3. miRNA Expression by Microarray

Total RNA was harvested using TRIzol (Invitrogen) and the miRNeasy mini kit (QIAGEN), according to manufacturer's instructions. RNA quantity was determined using a NanoDrop 1000 instrument. RNA was labeled using the miRCURY™ Hy3™/Hy5™ Power labeling kit and hybridized onto the miRCURY™ LNA Array (v.16.0). The slides were washed and scanned using an Axon GenePix 4000B microarray scanner.

Scanned images were imported into GenePix Pro 6.0 software (axon) for grid alignment and data extraction. miRNAs with intensity ≥50 in all samples were selected for calculating a normalization factor after averaging the replicated miRNAs. After normalization using median normalization, volcano plot filtering was used to identify significantly differentially expressed miRNAs. Hierarchical clustering was performed to visualize distinct miRNA expression profiling among samples.

### 2.4. miRNA-mRNA Coexpression Network Analysis

Network diagram analysis of miRNAs and mRNAs revealed regulatory relationships between miRNAs and their target genes. Target prediction information was derived from the following three resources: miRWalk (http://mirwalk.umm.uni-heidelberg.de/), miRDB (http://mirdb.org/), and TargetScan (http://www.targetscan.org/vert_60/). Predicted targets were filtered by identifying targets overlapping from the different prediction resources. miRNAs and predicted target genes were analyzed using Cytoscape software, and pathway enrichment was analyzed using the DAVID web resource (https://david.ncifcrf.gov/).

## 3. Statistical Methods

One-way analysis of variance was applied to assess differential expression of miRNAs between groups, and Benjamini–Hochberg FDR correction was applied, in addition to Tukey's honestly significant difference (HSD) post hoc test. A *p* value of <0.05 was considered to indicate a statistically significant difference.

## 4. Results

miRNA expression profiling was performed for NSCLC and SCLC samples with different RON expression levels. miRNA expression levels were more greatly influenced by tumor type than by RON expression levels.

**Figure 1:**
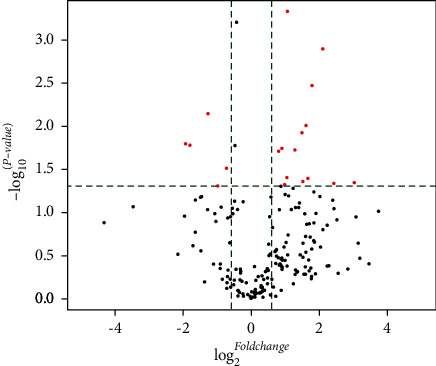
Differential expression of miRNAs in NSCLC and SCLC. Volcano plot of differentially expressed miRNAs between SCLC and NSCLC samples. The vertical lines correspond to 1.5-fold overexpression and underexpression, respectively. The horizontal line represents a *p* value of 0.05. The red points in the plot represents differentially expressed miRNAs which met the threshold for statistical significance.

**Figure 2:**
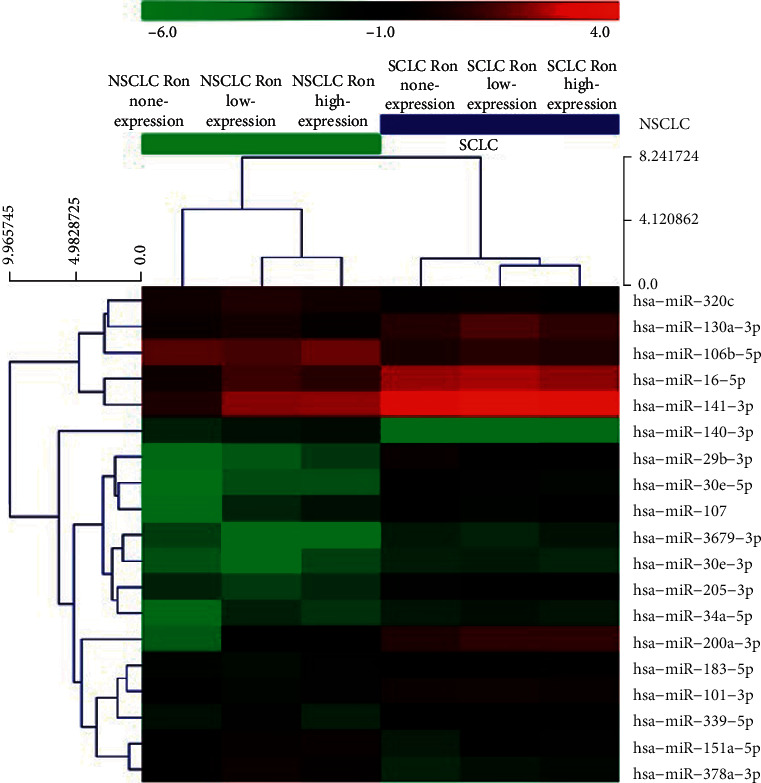
Heat map of miRNA expression in SCLC and NSCLC samples. The heat map diagram depicts the results of the two-way hierarchical clustering of miRNAs and sample type. Each row represents a miRNA and each column represents different sample type. The miRNA clustering tree is shown on the left, and the sample clustering tree appears at the top. The color scale shown at the top illustrates the relative expression level of a miRNA in the certain slide: red color represents a high relative expression level; green color represents a low relative expression level.

Comparison of miRNA expression between NSCLC and SCLC identified 85 significantly differentially expressed miRNA genes. Among these, 55 (65%) were overexpressed in SCLC vs. NSCLC, and 30 (35%) were underexpressed. miRNAs with fold-change with **≥**1.5-fold overexpression or **≤**0.5-fold underexpression are given in Table 1 and Figure 1. In addition, RON overexpression was associated with discrete miRNA expression patterns (Table 2), particularly in NSCLC patients (Table 3), compared to RON nonexpressing samples; hierarchical clustering was performed to show the distinct miRNA expression profiling among samples (Figure 2).

**Figure 3:**
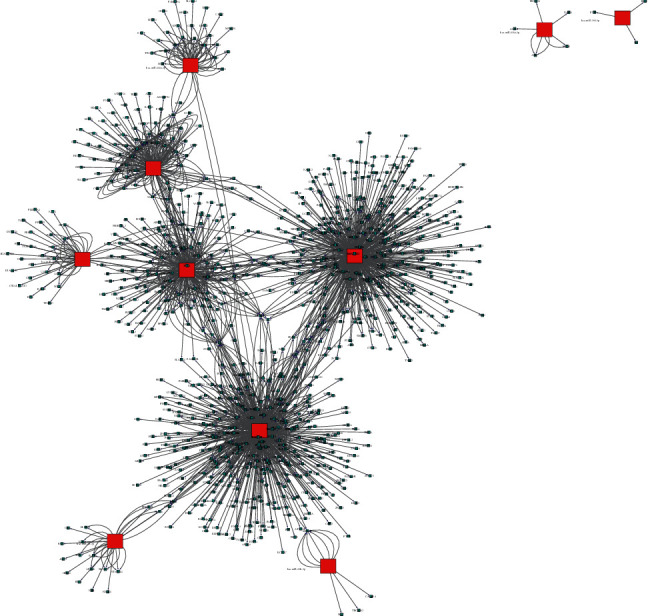
miRNA gene interaction network. Red boxes represent miRNAs, and blue square nodes represent the predicted target genes. Edges show the inhibitory effect of a miRNA to its predicted targets. Overexpressed miRNAs hsa-miR-1290 and hsa-miR-33a-5p show the most target mRNAs. hsa-miR-27b-3p and hsa-miR-21-5p have the highest degree of connectivity among underexpressed miRNAs. Degree refers to the contribution of one miRNA to the target genes around it or the contribution of one gene to the miRNAs around it. The key miRNAs and genes in the network always have the highest degrees of connectivity.

**Figure 4:**
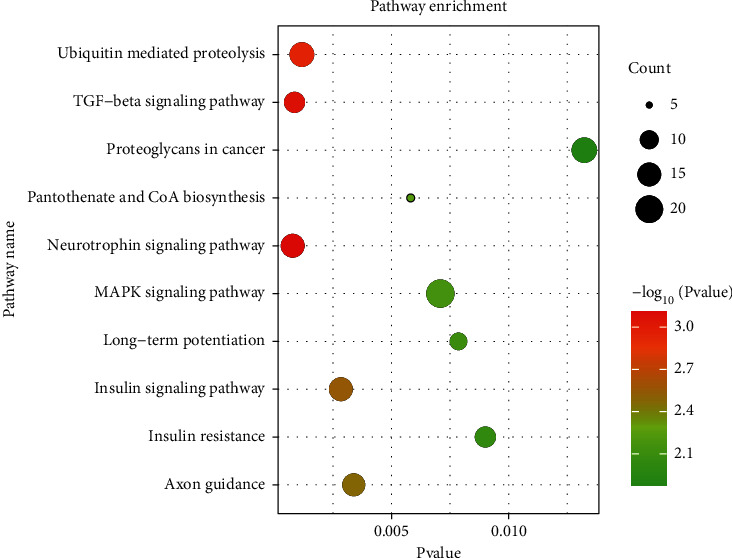
miRNA target pathway enrichment analysis. The top 10 significantly enriched pathways as predicted from target genes of differentially expressed miRNAs in RON overexpressing NSCLC samples.

**Figure 5:**
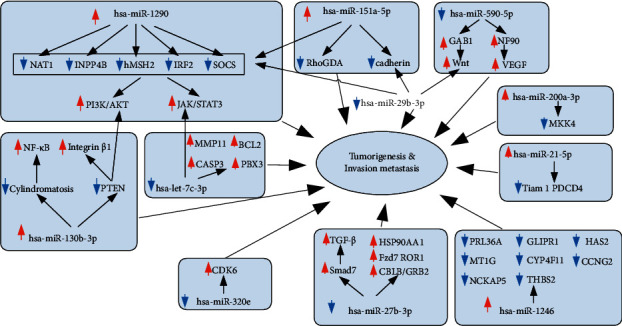
A network of RON-related miRNA target genes and potential mechanism of action. Upregulation or downregulation of a specific miRNA or target gene is represented by an upward (red) or a downward (blue) arrow, respectively.

In order to discern the major miRNA genes associated with RON overexpression in NSCLC, differentially expressed miRNA genes were obtained after comparing the RON high-expressing and RON nonexpressing NSCLC samples, and a gene coexpression network was constructed. Using the miRWalk, miRDB, and TargetScan miRNA target prediction databases, 1827 overlapping target genes were identified for the 10 most significantly differentially expressed miRNAs. A regulatory network between the overlapping target genes and their upstream miRNAs was constructed using Cytoscape (Figure 3). In order to identify the potential mechanism by which RON may promote NSCLC through miRNA-mediated regulation, the 1827 predicted target genes were then subjected to DAVID pathway enrichment analysis. This pathway enrichment revealed that RON-related miRNAs are predicted to target genes that significantly affect pathways involved in tumorigenesis. The top 4 significantly enriched signaling pathways were pathways in cancer, the PI3K-Akt signaling pathway, the MAPK signaling pathway, endocytosis, and proteoglycans in cancer (Figure 4). In addition, the top 10 overexpressed and top 10 underexpressed miRNAs and their major targets which might have key function were predicted from published data (Figure 5). Overexpression or underexpression of miRNAs detected by the microarray chip is represented by an upward (red) or a downward (blue) arrow, respectively. Changes of target genes expression levels, which were inversely correlated with the expression of the miRNAs that target them, are also represented by a down (blue) or up (red) arrow.

## 5. Discussion

Lung cancer is one of the most common cancers and a leading cause of cancer-related deaths. The identification of critical genetic factors leading to cellular transformation, including EGFR, ALK, and Ros1, has resulted in significant improvements in therapy for advanced lung cancer based on radiotherapy and chemotherapy and has improved the prognosis and quality of life for patients with lung cancer [1, 2].

The RON receptor tyrosine kinase is an oncogene that plays an important role in the development of lung cancer. RON overexpression can induce a complex genetic program that results in cell dissociation, migration, and extracellular matrix invasion, which may be important in several tumor types, including lung cancer. The extent of RON overexpression varies widely among different lung cancer cells and between different subtypes of lung cancers. The frequency and intensity of RON expression in different lung cancer subtypes is not well characterized, and the specific mechanism by which RON overexpression contributes to the pathogenesis of different lung cancer subtypes remains unknown. R. Kanteti et al. reported that RON expression in NSCLC was lower than in primary and secondary SCLC tumors [6]. Reports from M. Wang confirm that RON overexpression is common in lung adenocarcinoma, suggesting that RON may initiate oncogenic programs and plays an important role in the pathogenesis of lung adenocarcinoma [10, 11].

miRNAs play important roles in lung tumor progression. Using high-throughput RNA sequencing and bioinformatics, many tumor-related miRNAs and their predicted targets that are oncogenes or tumor suppressor genes have been reported. In this study, miRNA expression patterns in RON high-expressing and RON nonexpressing lung cancer samples were profiled, and we predicted potential gene-miRNA interactions with a systematic bioinformatics approach. Our study demonstrates that the type of tumors, such as SCLC or NSCLC, has the greatest impact on differential miRNA expression patterns. Within subgroups, RON expression had a greater impact on overexpression or underexpression of miRNAs in NSLCL samples than in SLCL samples.

46 differentially expressed miRNA genes in RON high-expressing NSCLC vs. RON nonexpressing NSCLC samples were identified; most of these miRNAs have reported functions in cancer. In addition, we also identified some putative genetic interactions involving networks of miRNAs, such as miR-33a-5p, hsa-miR-33b-5p, hsa-miR-31-5p, and miR-106a-5p. For example, miR-106a-5p has been reported to inhibit the migration and invasion of renal cell carcinoma through targeting PAK5. It has also been reported that miR-16a-5p acts as an onco-miRNA by targeting PTEN, E2F3, and E2F5. The Akt/mTOR and PI3K/AKT signaling pathways were also directly regulated by MiR-106a-5p [12, 13].

The most significantly overexpressed miRNA between RON high-expressing and RON nonexpressing NSCLC samples was hsa-miR-1290. hsa-miR-1290 directly targets a number of genes, including NAT1, INPP4B, SOCS4, IRF2, and hMSH2, and activates the JAK/STAT3 and PI3K/AKT signaling pathways. Inhibition of miR-1290 resulted in a decrease in stemness markers and EMT markers in NSCLC. Anti-miR-1290 treatment suppressed proliferation, sphere-formation, colony formation, and invasion of NSCLC cells in vitro [14, 15].

The overexpressed miRNA, hsa-miR-1246, is closely related to the occurrence and development of p53 family tumors. Inhibition of miR-1246 in NSCLC resulted in decreased stemness markers and EMT markers. Anti-miR-1246 treatment suppressed the proliferation, sphere-formation, colony formation, and invasion of NSCLC. Furthermore, genes repressed by miR-1246 include PRL36A, GLIPR1, HAS2, NCKAP5, MT1G, CYP4F11, CCNG2, and THBS2 [14, 16, 17].

miR-21-5p promotes peritoneal metastasis through EMT in gastric cancer, increases the proliferation, migration, and invasion of colon cancer by downregulating Tiam1, promotes cell migration and invasion in esophageal cancer by targeting PDCD4, induces metastasis of human cervical carcinoma cells, and can be used as a biomarker to predict the recurrence of digestive system tumors [18, 19].

hsa-miR-151a is overexpressed in primary NSCLC and induces proliferation, migration, and EMT as an onco-miRNA by targeting E-cadherin mRNA. hsa-miR-151a also promotes metastasis and functions synergistically with FAK to inhibit RhoGDIA. In addition, miR-151a-5p targets SOCS5 and activates downstream JAK2/STAT3 signaling [20, 21].

hsa-miR-130b, an onco-miRNA, is directly regulated by NF-*κ*B and sustains NF-*κ*B activation by decreasing cylindromatosis expression. hsa-miR-130b-3p also downregulates PTEN expression, which promotes the proliferation, migration, invasion, and cytoskeletal rearrangement through the activation of PI3K and integrin *β*1 signaling pathways. Moreover, miR-130b-3p inhibitors induced apoptosis [22–24].

hsa-miR-200a-3p regulates EMT-related gene expression, promotes the proliferation of cancer cells by posttranscriptionally regulating cytoplasmic collapsin response mediator, and prevents apoptotic cell death through downregulation of MKK4 [25].

The top downregulated RON overexpression-associated miRNA was hsa-let-7c-3p. hsa-let-7c-3p, a metastasis suppressor, was shown to suppress cell migration and invasion by downregulating K-RAS, MMP11, Bcl-2, CASP3, and PBX3. Moreover, hsa-let-7c-3p directly repressed the cisplatin-activated IL-6/STAT3 prosurvival pathway to modulate chemosensitivity, and transfection of hsa-let-7c restored sensitivity to cisplatin and increased the rate of esophageal squamous carcinoma cellular apoptosis after exposure [26, 27].

Increased expression of hsa-miR-31-5p inhibits cell proliferation, migration, and invasion by regulating the Sp1 transcription factor in hepatocellular carcinoma. However, the function of hsa-miR-31-5p was shown to differ, as hsa-miR-31-5p was also demonstrated to positively influence cell motility in correlation with metastatic status by regulating PGE2, which was mediated by EP1-ERK-MMP9 signaling. Moreover, comprehensive miRNA expression profiling analysis found that hsa-miR-31-5p is a diagnostic biomarker for pancreatic cancer [28, 29].

Hsa-let-7a expression plays an important role in tumorigenesis through repressing c-Myc and is significantly downregulated in a number of cancers, including hepatocellular cancer, breast cancer, and ovarian cancer [30].

The hsa-miR-320 family, particularly hsa-miR-320e, is downregulated in colorectal adenoma and affects colorectal tumor proliferation by targeting CDK6. hsa-miR-320e plays an important role in the growth of colorectal tumors and is considered as a biomarker for the early detection of colorectal tumors [31].

Upregulated hsa-miR-27b has a protective role in cell proliferation and migration by targeting Smad7 and affecting the TGF-*β* pathway. hsa-miR-27b-3p directly targets HSP90AA1 and Fzd7 in NSCLC, ROR1 in gastric cancer, and CBLB/GRB2 in breast cancer to suppresses cell proliferation, migration, invasion, and expression of MET [32, 33].

hsa-miR-29b-3p suppresses cell proliferation, migration, invasion, EMT, and metastasis in vitro and in vivo by regulating DNMT3B, PGRN, and STAT3, elevating E-cadherin expression, and decreasing Snail and vimentin. hsa-miR-29b-3p also represses Wnt signaling, TGF-*β*1 signaling, and STAT3 signaling [34, 35].

miR-590-5p inhibits breast cancer cell stemness and metastasis by targeting SOX2, inhibits colorectal cancer angiogenesis and metastasis by regulating the NF90/VEGFA axis, inhibits gastric cancer cell growth and chemosensitivity through the RECK and AKT/ERK pathway, inhibits growth of HepG2 cells via decrease of S100A10 expression and inhibition of Wnt pathway, and suppresses the proliferation and invasion of NSCLC by regulating GAB1 [36, 37].

RON high-expression activates or represses some transcription factors, which likely impacts the expression of a number of miRNAs. The 46 miRNAs that were differentially expressed miRNAs between RON high-expressing and RON nonexpressing NSCLC samples might be important factors that mediate the pathogenesis of NSCLC. These miRNAs promote tumor development via regulation of numerous targets, multiple signaling pathways, and biological functions and behaviors. Among these, EMT and invasion were identified as important functions enriched in these miRNAs, which is consistent with previous findings. Constitutively, high RON expression leads to morphological scattering or stabilized EMT and TGF-*β*1, and activation of the MAPK and Ras pathways was closely related to RON-mediated EMT. JAK/STAT, MAPK, and Ras signaling are possible common mediators of the functional behavior of these miRNAs, as the JAK/STAT signaling pathway mediates the biological effects of various external stimuli and controls survival, proliferation, and differentiation of several cell types. RON overexpression may be involved in the pathological process of tumorigenesis through these pathways, although interrogation of the specific mechanisms involved is beyond the scope of this study.

The miRNA expression between NSCLC and SCLC samples was clustered, and a heat map was constructed to visualize miRNA expression patterns. Our findings suggest that while RON expression levels are associated with distinct miRNA expression patterns in NSCLC, the specific lung cancer subtype (either NSCLC or SCLC) has a greater impact on miRNA expression profiles. Furthermore, we analyzed the interactions between miRNAs and their potential target genes that were differentially expressed based on differential RON expression in NSCLC, and we constructed a network of miRNA interactions using Cytoscape software. Our data suggest that the RON-associated miRNAs may impact a number of pathways, including PI3K-Akt, MAPK, endocytosis, proteoglycans in cancer, focal adhesion, and Ras signaling pathway-related activation state; these pathways may be differentially activated between NSCLC samples that exhibit distinct patterns of RON expression and are consistent with previous research which suggested that RON promotes oral squamous cell carcinoma progression by regulating EMT and the MAPK signaling pathway [38].

## 6. Conclusion

In summary, here, miRNA expression patterns of lung cancer associated with differential expression of the human receptor tyrosine kinase RON were profiled. We identified differentially expressed miRNA between NSCLC and SCLC, as well as between NSCLC samples with high RON expression and lacking RON expression. Our results suggest that miRNAs regulating PI3K-Akt, MAPK, endocytosis, proteoglycans in cancer, focal adhesion, and Ras signaling may exhibit significantly different expression patterns associated with differential RON expression in NSCLC. EMT and PI3K-Akt and MAPK signaling pathways are significantly regulated by RON expression levels. The approach and findings of this study may provide new perspectives on the tumor-promoting effect of RON and may lead to the development of improved diagnostic and therapeutic strategies for lung cancer. Further research will verify these differentially expressed miRNAs on larger tissue samples and then evaluate the miRNAs-regulated gene networks predicted by bioinformation analysis. Functional verification of gene-gene interactions can be carried out at both cellular and animal levels.

## Figures and Tables

**Table 1:** Differentially expressed miRNAs with ≥1.5-fold overexpression or ≤0.5-fold underexpression in SCLC vs. NSCLC samples.

ID	Name	Fold-change ≥1.5 SCLC vs. NSCLC	ID	Name	Fold-change 1.5 SCLC vs. NSCLC
11040	hsa-miR-29b-3p	8.051603	11260	hsa-miR-151a-5p	0.41632
28191	hsa-miR-30e-5p	5.304497	46228	hsa-miR-320c	0.59677
11000	hsa-miR-200a-3p	4.301609	42630	hsa-miR-140-3p	0.26368
10946	hsa-miR-141-3p	3.426177	19582	hsa-miR-106b-5p	0.50352
10923	hsa-miR-107	3.120694	148668	hsa-miR-378a-3p	0.28814
10967	hsa-miR-16-5p	3.002912			
148038	hsa-miR-3679-3p	2.81699			
145996	hsa-miR-205-3p	2.803615			
31026	hsa-miR-101-3p	2.425494			
10138	hsa-miR-130a-3p	2.058103			
10977	hsa-miR-183-5p	2.040765			
145676	hsa-miR-30e-3p	1.961315			
42739	hsa-miR-339-5p	1.858763			
27217	hsa-miR-34a-5p	1.760212			

**Table 2:** miRNAs with statistically significant differential expression ≥1.5-fold overexpression or ≤0.5-fold underexpression in high RON expressing lung cancer samples compared to RON nonexpressing samples.

ID	Name	Fold-change ≥ 1.5 overexpression in RON high-expressing vs. RON nonexpressing	ID	Name	Fold-change ≤ 0.5 underexpression in RON high-expressing vs. RON nonexpressing
145859	hsa-miR-33a-5p	2.36	28302	hsa-miR-27b-3p	0.49
148642	hsa-miR-1246	1.59	13138	Hy3	0.39
11000	hsa-miR-200a-3p	1.55			
10936	hsa-miR-130b-3p	1.55			
11260	hsa-miR-151a-5p	1.55			

**Table 3:** Differentially expressed miRNAs with >1.5-fold overexpression or 0.5-fold underexpression in high RON expressing NSCLC samples compared to RON nonexpressing NSCLC samples.

ID	Name	Fold-change ≥1.5 RON high-expressing vs. RON nonexpressing NSCLC	ID	Name	Fold-change ≤0.5 RON high-expressing vs. RON nonexpressing NSCLC
46921	hsa-miR-1290	7.57	13138	hsa-let-7c-3p	0.24
148642	hsa-miR-1246	7.28	147162	hsa-miR-31-5p	0.33
25611	spike_control_v2_19	4.1	147512	hsa-let-7a	0.34
11052	hsa-miR-21-5p	3.29	147506	Hy3	0.42
27318	spike_control_v2_23	3.2	28302	hsa-miR-320e	0.46
145859	hsa-miR-33a-5p	2.41	11040	hsa-miR-27b-3p	0.46
145950	hsa-miR-33b-5p	2.3	147691	hsa-miR-29b-3p	0.47
11260	hsa-miR-151a-5p	1.99	11030	hsa-miRPlus-A1015	0.47
10936	hsa-miR-130b-3p	1.89	46801	hsa-miR-106a-5p	0.49
11000	hsa-miR-200a-3p	1.52	17503	hsa-miR-590-5p	0.49

## Data Availability

The data used to support the findings of this study are available from the corresponding author upon request.

## References

[1] Pao W., Girard N. (2011). New driver mutations in non-small-cell lung cancer. *The Lancet Oncology*.

[2] Wolf J., Seto T., Han J.-Y. (2020). Capmatinib inMETExon 14-mutated orMET-amplified non-small-cell lung cancer. *New England Journal of Medicine*.

[3] Dustin D., Gu G., Beyer A. R. (2021). RON signalling promotes therapeutic resistance in ESR1 mutant breast cancer. *British Journal of Cancer*.

[4] Bourn J. R., Ruiz-Torres S. J., Hunt B. G., Benight N. M., Waltz S. E. (2021). Tumor cell intrinsic RON signaling suppresses innate immune responses in breast cancer through inhibition of IRAK4 signaling. *Cancer Letters*.

[5] Weng T.-H., Yao M.-Y., Xu X.-M. (2020). RON and MET Co-overexpression are significant pathological characteristics of poor survival and therapeutic targets of tyrosine kinase inhibitors in triple-negative breast cancer. *Cancer Research and Treatment*.

[6] Krishnaswamy S., Mohammed A. K., Tripathi G., Alokail M. S., Al-Daghri N. M. (2017). Splice variants of the extracellular region of RON receptor tyrosine kinase in lung cancer cell lines identified by PCR and sequencing. *BMC Cancer*.

[7] Croce C. M., Calin G. A. (2005). miRNAs, cancer, and stem cell division. *Cell*.

[8] Abd-Aziz N., Kamaruzman N. I., Poh C. L. (2020). Development of microRNAs as potential therapeutics against cancer. *Journal of oncology*.

[9] Yu P. T.-W., Lowy A. M., Aronow B. (2009). RON and c-met differentially regulate microRNA expression during pancreatic carcinogenesis. *Journal of the American College of Surgeons*.

[10] Chen Y.-Q., Zhou Y.-Q., Fisher J. H., Wang M.-H. (2002). Targeted expression of the receptor tyrosine kinase RON in distal lung epithelial cells results in multiple tumor formation: oncogenic potential of RON in vivo. *Oncogene*.

[11] Chen Y.-Q., Zhou Y.-Q., Fu L.-H., Wang D., Wang M.-H. (2002). Multiple pulmonary adenomas in the lung of transgenic mice overexpressing the RON receptor tyrosine kinase. *Carcinogenesis*.

[12] Pan Y.-J., Wei L.-L., Wu X.-J., Huo F.-C., Mou J., Pei D.-S. (2017). MiR-106a-5p inhibits the cell migration and invasion of renal cell carcinoma through targeting PAK5. *Cell Death & Disease*.

[13] Dong S., Zhang X., Liu D. (2019). Overexpression of long noncoding RNA GAS5 suppresses tumorigenesis and development of gastric cancer by sponging miR-106a-5p through the Akt/mTOR pathway. *Biology Open*.

[14] Zhang W. C., Chin T. M., Yang H. (2016). Tumour-initiating cell-specific miR-1246 and miR-1290 expression converge to promote non-small cell lung cancer progression. *Nature Communications*.

[15] Kim G., An H.-J., Lee M.-J. (2016). Hsa-miR-1246 and hsa-miR-1290 are associated with stemness and invasiveness of non-small cell lung cancer. *Lung Cancer*.

[16] Bhagirath D., Yang T. L., Bucay N. (2018). microRNA-1246 is an exosomal biomarker for aggressive prostate cancer. *Cancer Research*.

[17] Cooks T., Pateras I. S., Jenkins L. M. (2018). Mutant p53 cancers reprogram macrophages to tumor supporting macrophages via exosomal miR-1246. *Nature Communications*.

[18] Zhang R., Xia T. (2017). Long non-coding RNA XIST regulates PDCD4 expression by interacting with miR-21-5p and inhibits osteosarcoma cell growth and metastasis. *International Journal of Oncology*.

[19] He J.-H., Li Y.-G., Han Z.-P. (2018). The CircRNA-ACAP2/hsa-miR-21-5p/Tiam1 regulatory feedback circuit affects the proliferation, migration, and invasion of colon cancer SW480 cells. *Cellular Physiology and Biochemistry*.

[20] Sanders K. (2016). *Novel Roles for miR-151a and miR-128 in Cancer*.

[21] Daugaard I., Sanders K. J., Idica A. (2017). miR-151a induces partial EMT by regulating E-cadherin in NSCLC cells. *Oncogenesis*.

[22] Cui X., Kong C., Zhu Y. (2016). miR-130b, an onco-miRNA in bladder cancer, is directly regulated by NF-*κ*B and sustains NF-*κ*B activation by decreasing Cylindromatosis expression. *Oncotarget*.

[23] Shui Y., Yu X., Duan R. (2017). miR-130b-3p inhibits cell invasion and migration by targeting the Notch ligand Delta-like 1 in breast carcinoma. *Gene*.

[24] Shakespear N., Ogura M., Yamaki J., Homma Y. (2020). Astrocyte-derived exosomal microRNA miR-200a-3p prevents MPP+-Induced apoptotic cell death through down-regulation of MKK4. *Neurochemical Research*.

[25] Zhou D., Zhang L., Sun W. (2017). Cytidine monophosphate kinase is inhibited by the TGF-*β* signalling pathway through the upregulation of miR-130b-3p in human epithelial ovarian cancer. *Cellular Signalling*.

[26] Wang L., Li J., Li Y., Pang L.-B. (2020). Hsa-let-7c exerts an anti-tumor function by negatively regulating ANP32E in lung adenocarcinoma. *Tissue and Cell*.

[27] Sugimura K., Miyata H., Tanaka K. (2012). Let-7 expression is a significant determinant of response to chemotherapy through the regulation of IL-6/STAT3 pathway in esophageal squamous cell carcinoma. *Clinical Cancer Research*.

[28] Mlcochova J., Faltejskova-Vychytilova P., Ferracin M. (2015). microRNA expression profiling identifies miR-31-5p/3p as associated with time to progression in wild-type RAS metastatic colorectal cancer treated with cetuximab. *Oncotarget*.

[29] Zhao G., Han C., Zhang Z., Wang L., Xu J. (2017). Increased expression of microRNA-31-5p inhibits cell proliferation, migration, and invasion via regulating Sp1 transcription factor in HepG2 hepatocellular carcinoma cell line. *Biochemical and Biophysical Research Communications*.

[30] Liu Y., Yin B., Zhang C., Zhou L., Fan J. (2012). Hsa-let-7a functions as a tumor suppressor in renal cell carcinoma cell lines by targeting c-myc. *Biochemical and Biophysical Research Communications*.

[31] Tadano T., Kakuta Y., Hamada S. (2016). microRNA-320 family is downregulated in colorectal adenoma and affects tumor proliferation by targeting CDK6. *World Journal of Gastrointestinal Oncology*.

[32] Tao J., Zhi X., Zhang X. (2015). miR-27b-3p suppresses cell proliferation through targeting receptor tyrosine kinase like orphan receptor 1 in gastric cancer. *Journal of Experimental & Clinical Cancer Research*.

[33] Zeng X., Huang C., Senavirathna L., Wang P., Liu L. (2017). miR-27b inhibits fibroblast activation via targeting TGF*β* signaling pathway. *BMC Cell Biology*.

[34] Lv M., Zhong Z., Huang M., Tian Q., Jiang R., Chen J. (2017). lncRNA H19 regulates epithelial-mesenchymal transition and metastasis of bladder cancer by miR-29b-3p as competing endogenous RNA. *Biochimica et Biophysica Acta (BBA) - Molecular Cell Research*.

[35] Ding D., Li C., Zhao T., Li D., Yang L., Zhang B. (2018). LncRNA H19/miR-29b-3p/PGRN axis promoted epithelial-mesenchymal transition of colorectal cancer cells by acting on Wnt signaling. *Molecules and Cells*.

[36] Zhou Q., Zhu Y., Wei X. (2016). MiR-590-5p inhibits colorectal cancer angiogenesis and metastasis by regulating nuclear factor 90/vascular endothelial growth factor A axis. *Cell Death & Disease*.

[37] Ekhteraei‐Tousi S., Mohammad‐Soltani B., Sadeghizadeh M., Mowla S. J., Parsi S., Soleimani M. (2015). Inhibitory effect of hsa‐miR‐590‐5p on cardiosphere‐derived stem cells differentiation through downregulation of TGFB signaling. *Journal of Cellular Biochemistry*.

[38] Kim S. A., Lee K. H., Lee D. H. (2019). Receptor tyrosine kinase, RON, promotes tumor progression by regulating EMT and the MAPK signaling pathway in human oral squamous cell carcinoma. *International Journal of Oncology*.

